# A POSHE-Based Optimum Clip-Limit Contrast Enhancement Method for Ultrasonic Logging Images

**DOI:** 10.3390/s18113954

**Published:** 2018-11-15

**Authors:** Qingqing Fu, Zhengbing Zhang, Mehmet Celenk, Aiping Wu

**Affiliations:** 1 Electronics and Information School, Yangtze University, Jingzhou 434023, China; jpufqq@yangtzeu.edu.cn (Q.F.); wuaping@yangtzeu.edu.cn (A.W.); 2 National Demonstration Center for Experimental Electrical & Electronic Education, Yangtze University, Jingzhou 434023, China; 3 School of Electrical Engineering and Computer Science, Ohio University, Athens, OH 45701, USA; celenk@ohio.edu

**Keywords:** optimum clip-limit, partially overlapped sub-block histogram-equalization (POSHE), contrast enhancement, ultrasonic logging image

## Abstract

Enabled by piezoceramic transducers, ultrasonic logging images often suffer from low contrast and indistinct local details, which makes it difficult to analyze and interpret geologic features in the images. In this work, we propose a novel partially overlapped sub-block histogram-equalization (POSHE)-based optimum clip-limit contrast enhancement (POSHEOC) method to highlight the local details hidden in ultrasonic well logging images obtained through piezoceramic transducers. The proposed algorithm introduces the idea of contrast-limited enhancement to modify the cumulative distribution functions of the POSHE and build a new quality evaluation index considering the effects of the mean gradient and mean structural similarity. The new index is designed to obtain the optimal clip-limit value for histogram equalization of the sub-block. It makes the choice of the optimal clip-limit automatically according to the input image. Experimental results based on visual perceptual evaluation and quantitative measures demonstrate that the proposed method yields better quality in terms of enhancing the contrast, emphasizing the local details while preserving the brightness and restricting the excessive enhancement compared with the other seven histogram equalization-based techniques from the literature. This study provides a feasible and effective method to enhance ultrasonic logging images obtained through piezoceramic transducers and is significant for the interpretation of actual ultrasonic logging data.

## 1. Introduction

**Figure 1:**
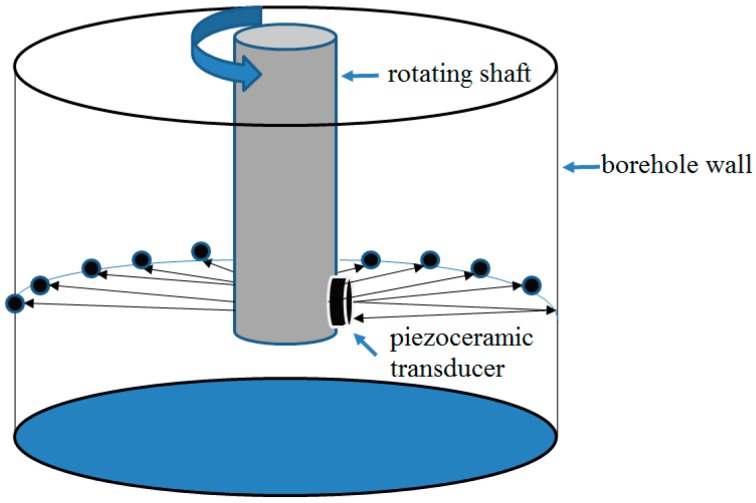
Diagram of the operating principle.

In the past decades, piezoceramic transducers with the unique capacity of actuating and sensing in a wide-frequency band [1,2,3,4,5], play important roles in ultrasonic techniques, enabling structural health monitoring (SHM) [6,7,8,9] and nondestructive testing (NDT) [10,11,12,13] in many fields [14,15,16,17], and one of them is oil and gas exploration [18,19,20]. Piezoceramic transducers are often used to generate and detect ultrasonic waves [21,22,23,24] that travel through different mediums with reflection and refraction, based on which images of mediums can be obtained [25,26,27,28,29,30], and this practice is commonly used in well logging [31,32,33]. In the process of ultrasonic imaging logging, acoustic imaging instruments use a rotating piezoceramic transducer to emit and receive ultrasonic pulses traveling through borehole fluid. The acoustic wave amplitude attenuation and the acoustic wave travel time are both recorded and converted into color images. The acoustic travel time and amplitude attenuation signals can reflect characteristics about borehole shape and variation in rock properties. Further, the two types of signals are visualized as 360° images of the borehole wall [34,35,36]. The diagram of the operating principle is illustrated in Figure 1. The transducer consists of several piezoceramic plates in parallel, and the material of the acoustic energy transducer used for the piezoceramic plates is Lead Zirconate Titanate (PZT), which has a strong piezoelectric effect. The frequency, voltage coefficient, electromechanical coupling coefficient, static capacitance and working temperature are the most important parameters with which we are concerned. The frequency of operation depends on different acoustic logging methods, and its value range from KHz to MHz. Due to the advantages of covering the entire borehole wall with the intuitive image, ultrasonic well logging images have a wide range of applications in the field of geophysical exploration [34,35,36,37,38,39,40], such as borehole shape analysis, evaluation on borehole breakouts [36], casing corrosion inspection in cased hole wells [37], identification of fractures and breakout regions around the wellbore and estimation of tectonic stress fields [36,38]. The piezoceramic-enabled ultrasonic well logging images are also used to analyze the geological structures for deep mineral prospecting [39] and generate a porosity spectrum of carbonate reservoirs [40]. Since the logging images carry important information, their quality greatly influences the interpretation of geological information. However, impacted by the logging instrument and down hole environment, the gray level distribution of captured images often exhibits a very narrow dynamic range (low contrast) [41,42]. In this case, some details reflecting the geological features within the image cannot be observed clearly, which brings difficulties in understanding and interpreting the geological target characteristics. There are two ways to solve the problem. One strategy is to optimize the hardware, such as using composite materials to improve the sensitivity of the transducer and using a phased-arc array transmitter with azimuthal detection capability to improve the acquisition accuracy [43,44]. The other way is based on signal processing methods to improve the captured images. In this paper, to accomplish a better visual quality improvement for ultrasonic logging images, our discussion mainly focuses on contrast enhancement methods in the field of image processing.

Histogram equalization (HE) is considered one of the most popular techniques to improve contrast because of its simplicity and ease of implementation. It generates a uniform output histogram by means of stretching the dynamic range of the input image histogram, thereby improving the image contrast [45,46]. However, HE often produces undesirable artifacts because of over enhancement when a few consecutive gray levels occupy substantial areas in an image and considerably changes the mean brightness of the input image [45,46,47,48,49]. In addition, HE may also cause loss of details since some gray levels with a smaller proportion pixel number are combined to form a certain gray level [49]. To avoid shortcomings of the classic HE, many techniques and improved algorithms on the basis of histogram equalization have been proposed in the past and have been widely utilized in the field of image enhancement [41,42,45,46,47,48,49,50,51,52,53,54,55,56,57,58,59,60,61,62,63,64,65,66,67,68,69,70,71,72,73,74,75]. Since preservation of the original brightness is crucial to avoid undesirable artifacts, the brightness preserving bi-histogram equalization (BBHE) [50] is developed to preserve the original brightness to a certain extent by individually equalizing two sub-histograms based on the mean value of the images. To obtain a higher degree of preservation, contrast enhancement using recursive mean-separate histogram equalization for scalable brightness preservation (RMSHE) [51] is presented and the mean brightness of the original input image is preserved to some extent by iteratively applying BBHE to the sub-histograms. When the recursion level becomes excessively large, the output mean eventually converges to the input mean. Nevertheless, it is hard to determine the optimal recursion level. Minimum mean brightness error bi-histogram equalization (MMBEBHE) [52], the extension of BBHE, was presented by Chen and Ramli to provide maximum brightness preservation. As it focuses on preservation of mean brightness, its contrast enhancement capability is not notable. An exposure based sub-image HE algorithm (ESIHE) [53] for low exposure gray scale image is presented by Singh and Kapoor, in which the histogram is split into over-exposed and under-exposed areas based on the image exposure thresholds. Further, Singh and Kapoor also proposed extensions of the ESIHE, referred to as RS-ESIHE [54]. The RS-ESIHE method is designed to perform the separation of image histogram recursively based on sub-histograms’ respective exposure thresholds, and each sub-histogram is then equalized individually. Clipping of the histogram is utilized in ESIHE and RS-ESIHE methods to avoid excessive enhancement.

The recursive division algorithms may not yield natural-looking results owing to inappropriate sub-divisions. In order to overcome the above-mentioned shortcomings, a contrast-enhancement approach called entropy-based dynamic sub-histogram equalization (EDSHE) [55] is proposed. It provides a stopping criterion to find the optimal division number of sub-histograms by recursively segmenting the histogram based on the entropy. There is no need to set any parameter and specify the level of divisions in this approach. Yet, the EDSHE method is at cost of calculating the entropy of sub-histogram many times. However, enhancement methods based on histogram division fail to expand some sections of the histogram. Thus, dynamic histogram equalization (DHE) [56] splits the image histogram by searching local minima and assigns specific gray level ranges for each sub-histogram before equalizing them individually. It can enhance the image without the loss of details. In recent years, the methods based on the histogram modification for controlling the enhancement effect have been proposed, such as adaptive gamma correction with weighting distribution (AGCWD) [57], histogram modification framework (HMF) [58], gradient and intensity histogram equalization (GIHE) [59], among others. AGCWD smoothens the fluctuant problem by means of the weighting distribution function and thus restricts the over enhancement of the gamma correction. Nevertheless, AGCWD tends to result in a loss of details in the bright areas of the input image if high peaks appear in the input histogram. AGCWD is suitable for the dimmed images. For the HMF method, the modified histogram is regarded as a solution to solve the optimization problem that minimizes a cost function. Penalty terms are incorporated into the optimization problem to adjust the level of contrast enhancement. GIHE makes use of gradient and intensity information of the image to modify the histogram. Thus, it alleviates over-enhancement.

Although these above discussed global techniques are appropriate to enhance the content of the whole input image, the power of highlighting details of the local regions is relatively low [48]. Nevertheless, local image enhancement is also needed in some applications including medicine, surveillance, remote sensing and consumer electronics [60]. A local histogram equalization (LHE)-based method is subsequently developed, which can also be termed a block-overlapped histogram equalization (BOHE) [61,62]. In this method, a rectangular sub-block of the input image is first defined and the center pixel of the region is updated using this histogram-equalized function of the current sub-block. The rectangular region is then moved pixel by pixel; then, the histogram equalization is repeated so as to obtain high contrast for all locations in the image. However, since LHE must be operated on each pixel for the whole image, the computation complexity is considerably high. Non-overlapped block histogram equalization (NOBHE) [60,62] is often used to reduce computation, but this method usually produces undesirable blocking effects. To address the above-mentioned limitation, Kim, et al. presented partially overlapped sub-block histogram-equalization (POSHE) [63], which can produce a similar contrast enhancement effect with BOHE and greatly reduce computation complexity and eliminate blocking effects. However, similar to other LHE methods, POSHE tends to generate an over-enhancement phenomenon, leading to noise amplification in partial regions. In order to overcome the noise amplification and blocking effects, contrast-limited adaptive histogram equalization (CLAHE) [64,65] was proposed, which first divides the image into many non-overlapped sub-blocks, then enhances each sub-block individually and finally employs an interpolation scheme to reduce the blocking effects. It is necessary to adjust the size of the sub-block and the clip-limit value in CLAHE. This method also needs to eliminate blocking effects, which make the method more complicated for implementation. For the same purpose, the multiple layers block overlapped histogram equalization (MLBOHE) [60] method is developed, which successfully overcomes noise amplification and intensity distortion problems. Unfortunately, the enhanced image is blurred to some extent since the median filters are adopted to reduce the noise.

Yan et al. [66] used the idea of sub-histogram equalization within a local window to process the logging images and then eliminate the blocking effects using morphing technology effectively. This method increases computation complexity due to introducing morphing technology. Tu et al. [42] enhanced ultrasonic logging images using the modified BOHE method, in which the data of the local depth range for the well is used to calculate the histogram, and the data are updated line by line. In a similar way, Halliburton’s XRMI (Extended Range Micro Imager) images are dynamically enhanced to improve the distinguishing ability of geological information in the literature [67]. This approach can achieve better results than the global histogram equalization algorithm in enhancing local details of image. However, it results in a small amount of noise amplification and over enhancement in the smooth region. Fu et al. [41] presents a novel image enhancement method based on CLAHE (CLAHE-PL) for ultrasonic logging images; the clip-limit value and power-law transformation parameters need to be adjusted to obtain desired results. We can see from the literature survey, it still remains a less explored area and challenge to enhance contrast, highlight the local details of the ultrasonic well logging images and achieve naturally looking results without excessive enhancement and undesirable artifacts. Therefore, a novel POSHE-based optimum clip-limit contrast enhancement method for ultrasonic logging images (POSHEOC) is proposed in this paper. The algorithm introduces the idea of contrast-limited enhancement to modify the cumulative distribution functions of the POSHE. The clip-limit value is a key issue influencing the enhancement effect. Hence, to obtain optimal clip-limit value for histogram equalization of the sub-block, a new quality evaluation index is devised considering the effects of the mean gradient and mean structural similarity. It selects the optimal clip-limit automatically according to different image data. In summary, our proposed method in this paper aims to enhance contrast, reveal the small and local details hidden in the ultrasonic logging images while preserving the naturalness of the original image without excessive enhancement and undesirable artifacts.

The rest of the paper is organized as follows. Section 2 discusses the traditional histogram equalization and POSHE. Section 3 presents our proposed POSHEOC contrast enhancement method in detail. Section 4 shows some experimental results and discussion of applying POSHEOC and other existing approaches to different ultrasonic logging images and Section 5 concludes the paper.

## 2. Related Work

This section covers the details of previous work which is related to the proposed POSHEOC method, including histogram equalization and POSHE; the discussion is based on the literature [62,68,69] and [63], respectively.

### 2.1. Histogram Equalization

The basic idea of histogram equalization is to transform the histogram of the original image into a uniform distribution. In this way, the dynamic range of gray value is broadened, thus achieving the effect of enhancing the overall contrast of the input image. In histogram equalization, assume that the input image has *L* gray levels. If *r_k_* is used to represent the *k*th gray level, then the *L* gray levels can be described as {*r*_0_, *r*_1_, ..., *r_L−_*_1_}; the probability density function (PDF) corresponding to the gray level distribution of the original image, *p*(*r_k_*), is given by

(1) $$p(r_{k})=\frac{N_{k}}{N},\;\;for\;k=0,1,\ldots ,L-1$$

where *N_k_* and *N* represent the number of pixels with gray level *r_k_* and the total number of pixels of the input image *X*, respectively. Then, the cumulative distribution function (CDF), *c*(*r_k_*), is calculated from the original PDF as follows:

(2) $$c(r_{k})=\sum_{j=0}^{k}p(r_{j})\;\;for\;k=0,1,\ldots ,L-1$$

Histogram equalization is a methodology that maps the input image into the whole dynamic range (*r*_0_, *r_L−_*_1_) using the CDF as a transformation function. The formula of the transformation function *f*(*r_k_*) is given as:

(3) $$f(r_{k})=r_{0}+(r_{L-1}-r_{0})\cdot c(r_{k})\;\;for\;k=0,1,\ldots ,L-1$$

Let *Y* represent the output image produced by histogram equalization; it can be given by the following expression:

(4) Y={Y(i,j)}={f(X(i,j))}

where *X*(*i*, *j*) represents the gray level at the spatial coordinates (*i*, *j*) for a specific pixel in the image, and *X*(*i*, *j*) Є *X*.

### 2.2. POSHE

To obtain local higher contrast and lessen the computation complexity in the process of local histogram equalization, partially overlapped sub-block histogram equalization (POSHE) is proposed [63] as a well-known LHE-based algorithm. Using POSHE, the computational complexity can be reduced while obtaining the same advantage of high contrast enhancement effects for all local regions as BOHE. Further, the common blocking effect problem accompanied with NOBHE can be reduced or eliminated. Hence, POSHE can be normally regarded as a synthesis of NOBHE and BOHE. The main idea behind this method is to move the sub-block at a certain step size, and this strategy results in the sub-block being partially overlapped. We then perform histogram equalization for the current sub-block. Finally, the transformation function for the overlapped areas can be generated by calculating the weighted sum of neighboring sub-blocks’ histograms equalization.

The specific steps of POSHE, which have been established based on Ref. [63], are given as follows.

*Step* *1:* Let us define an image with size *M* × *N*.
*Step* *2:* Assign an *m* × *n* sub-block at the top left corner. For computational simplicity, the size of the sub-block is selected to be equal to the quotient of the input image size divided by a multiple of two.
*Step* *3:* Perform local histogram equalization for the current sub-block.
*Step* *4:* The sub-block moves from left to right and from top to bottom by the horizontal step size and the vertical step size. Repeat *Step 3* until POSHE covers the entire input image plane.
*Step* *5:* After sub-block histogram equalization is completed, because each pixel is obviously histogram equalized more than once, accumulated equalization results on each pixel can be divided by its histogram equalization frequency and then produce each pixel value in the output image array.

**Figure 2:**
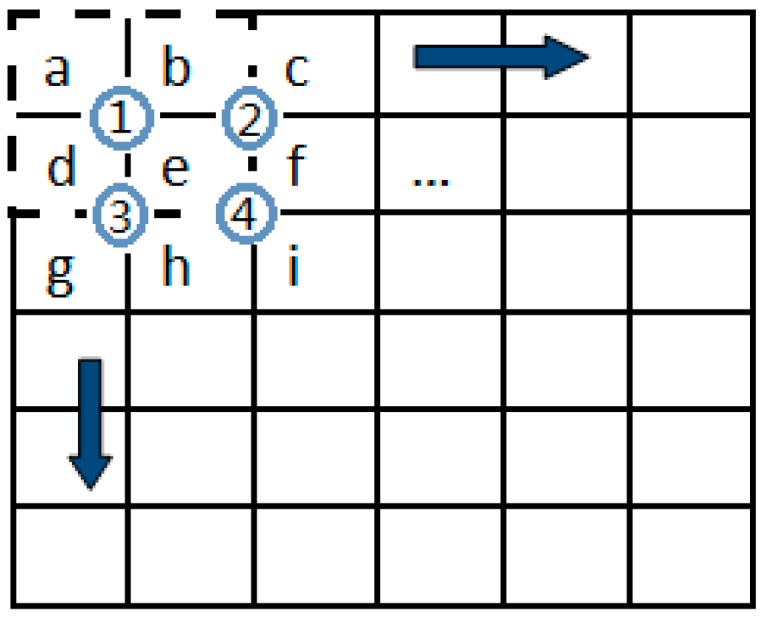
Example of POSHE.

To make the procedure more understandable, Figure 2 provides an example of POSHE. Assume the sub-block (*m = n*) is the area surrounded by a dashed box, as shown in Figure 2. The step size is selected to be a half of the sub-block size (*m*/2). The sub-block moves in the direction of the arrow for the regions a, b, c, d, e, f, g, h and i, which are composed of four partially overlapped sub-blocks. They are sub-block 1 (a, b, d, e, formed), sub-block 2 (b, c, e, f, formed), sub-block 3 (d, e, g, h, formed), and sub-block 4 (e, f, h, i, formed). The four sub-blocks are processed using POSHE with a step size of *m*/2. Suppose that the histogram equalization functions for each sub-block are $T_{1}(r_{k})$, $T_{2}(r_{k})$, $T_{3}(r_{k})$ and $T_{4}(r_{k})$, respectively. The sub-region *e* is the histogram equalized by four sub-blocks 1, 2, 3 and 4. It is obvious that the pixels in sub-region *e* are processed four times during this procedure. Then, the POSHE transformation function of the sub-region *e* can be defined as follows:

(5) $$s_{k}{}^{e}=\frac{1}{4}[T_{1}(r_{k}{}^{e})+T_{2}(r_{k}{}^{e})+T_{3}(r_{k}{}^{e})+T_{4}(r_{k}{}^{e})]$$

where $s_{k}{}^{e}$ is the gray values after POSHE for the pixels with value of *k* in the region *e*, and $r_{k}{}^{e}$ is the pixels with the value of *k* in the region *e*. Each histogram equalization function can be described as

(6) $$\begin{array}{l} T_{1}(r_{k}{}^{e})=\sum_{j=0}^{k}p_{1}(r_{j})\\ p_{1}(r_{j})=\frac{n_{j}{}^{1}}{\frac{4n}{9}}=\frac{n_{j}{}^{a}+n_{j}{}^{b}+n_{j}{}^{d}+n_{j}{}^{e}}{\frac{4n}{9}} \end{array}$$

(7) $$\begin{array}{l} T_{2}(r_{k}{}^{e})=\sum_{j=0}^{k}p_{2}(r_{j})\\ p_{2}(r_{j})=\frac{n_{j}{}^{2}}{\frac{4n}{9}}=\frac{n_{j}{}^{b}+n_{j}{}^{c}+n_{j}{}^{e}+n_{j}{}^{f}}{\frac{4n}{9}} \end{array}$$

(8) $$\begin{array}{l} T_{3}(r_{k}{}^{e})=\sum_{j=0}^{k}p_{3}(r_{j})\\ p_{3}(r_{j})=\frac{n_{j}{}^{3}}{\frac{4n}{9}}=\frac{n_{j}{}^{d}+n_{j}{}^{e}+n_{j}{}^{g}+n_{j}{}^{h}}{\frac{4n}{9}} \end{array}$$

(9) $$\begin{array}{l} T_{4}(r_{k}{}^{e})=\sum_{j=0}^{k}p_{4}(r_{j})\\ p_{4}(r_{j})=\frac{n_{j}{}^{4}}{\frac{4n}{9}}=\frac{n_{j}{}^{e}+n_{j}{}^{f}+n_{j}{}^{h}+n_{j}{}^{i}}{\frac{4n}{9}} \end{array}$$

where *n* denotes the number of pixels in the full region, $n_{j}{}^{x}$ represents the number of pixels in sub-region *x* with *j*th level, and *x* = a, b, …, i.

## 3. The Proposed Contrast Enhancement Method

The main purpose of our proposed algorithm is to improve contrast, enhance detail information hidden in the ultrasonic logging images, and achieve naturally looking results while reducing the over-enhancement and undesirable artifact effects. From the above analysis, if HE is directly used in the POSHE, over-enhancement problem will occur in the equalized sub-block image. This issue is resolved by limiting the contrast as indicated in the description of the CLAHE [64,65] method. The slope of the histogram equalization function related to the gray level distribution is confined. This goal can be achieved by allowing only a maximum number of pixels for each gray level. After clipping of the histogram is completed, in order to make the total histogram count identical to original number, the pixels that were clipped are evenly redistributed over the whole histogram, and therefore produce limited contrast enhancement. Inspired by the CLAHE method, we introduced the idea of contrast-limited enhancement to the POSHE and proposed a novel POSHE-based optimum clip-limit contrast enhancement method for improving ultrasonic logging images (POSHEOC). The clip-limit value is a key factor to control the enhancement effect. Hence, the mean gradient and mean structural similarity are taken into account to build a new quality evaluation index, which is used to obtain optimal clip-limit value for histogram equalization of the sub-block. It selects the optimal clip-limit automatically according to different image data. This proposed POSHEOC method consists of two main steps, namely, clipped histogram equalization and optimum clip-limit strategies. The description of each step is presented in detail in the following subsections.

### 3.1. Clipped Histogram Equalization

The POSHE algorithm employs the conventional histogram equalization (HE) algorithm to process each sub-block. Although the classic HE may provide the best visual performance under certain conditions, its major drawback is over-enhancement when high peaks occur in the PDF of the histogram for an image. As given in Equation (2) in Section 2.1, the enhancement from histogram equalization is heavily dependent on CDF. This is to say, if the curve of CDF is too steep, the HE will stretch the dynamic range of the high concentration regions excessively and will result in over-enhancement phenomenon. On the other hand, if the region of interest in the image occupies only a small percentage, it will not be appropriately enhanced. Therefore, the enhancement degree is proportional to the slope of *c*(*r_k_*) (defined in Section 2.1). The slope of *c*(*r_k_*) is given by

(10) $$\frac{d}{dr_{k}}c(r_{k})=p(r_{k})$$

The enhancement rate can be adjusted by reducing or increasing the value of *p*(*r_k_*). In order to limit the contrast to a desired level, a technique proposed in the CLAHE method [64,65] is restraining the slope of CDF for the original image, and it can be achieved by clipping the histogram with values over a predefined threshold, and the residual is redistributed uniformly to the histogram. The maximum slope is limited using a clip limit *β* to clip all histograms; *β* can be defined as follows [64,70]:

(11) $$\beta =\frac{N_{t}}{L_{t}}(1+\frac{\alpha }{100}(s_{\max}-1))$$

where *N_t_* and *L_t_* are total numbers of pixels and gray-levels in each region, respectively, $s_{\max}$ is the maximum allowable slope, and *a* is the clip factor. If α = 0, the minimum clip limit *β* is equal to ($\frac{N_{t}}{L_{t}}$) which can reach the maximum value of ($s_{\max}\cdot \frac{N_{t}}{L_{t}}$) for α = 100. It is clear that various $s_{\max}$ values will affect the processed results. Normally, $s_{\max}$ is set to four for the application of still X-ray images as reported in Ref. [64]. However, for other applications, it is recommended that a good selection for $s_{\max}$ is obtained by practical experiment.

When the POSHE algorithm is used, the histogram should be clipped before sub-block equalization and then evenly distributed to each gray-level. By limiting the histogram height, the slope of the CDF curve can be reduced, that is, the contrast enhancement can be reduced to restrain the noise amplification and local over-enhancement. The clipping and redistribution processes are described in the following steps:

*Step 1*: Define *h*(*k*) as the pixel number of gray-level *k* (*k* = 0, 1, …, *L_t_* − 1) and set the average number of pixels per grayscale as *N_av_* for any sub-block with size *m* × *n.* Then, $N_{t}=m\cdot n$, for an 8-bit grayscale image, *L_t_* = 256, and $N_{av}=\frac{N_{t}}{L_{t}}=\frac{m\times n}{256}$. The total number of pixels whose histogram values exceed the clip limit *β* is represented as *Excess* with the initial value being 0.

*Step 2*: Clip the histogram with the value beyond the clip limit *β*; i.e., if *h*(*k*) > *β*, *k* = 0, 1, …, *L_t_* − 1, then, *Excess* = *Excess* – *β* + *h*(*k*), *h*(*k*) = *β*. This step modifies the histogram by preserving the histograms that are less than or equal to *β*, while clipping the ones that exceed *β*.

*Step 3*: Accumulate exceeded pixel counts *Excess* obtained from *Step 2* and redistribute them to all the histogram bins. If *N_m_* is used to represent the number of pixels that should be equally assigned to each gray-level, then *N_m_* = *Excess*/*L_t_*. The histogram *h*(*k*) after the redistribution is given by Equation (12), and *Excess* after the redistribution is generated as in Equation (13):

(12) $$\begin{array}{l} h(k)=\left\{ \begin{array}{ll} h(k)+N_{m} & h(k)<\beta -N_{m}\\ \beta & \beta -N_{m}<h(k)<\beta \end{array}\right. \end{array}$$

(13) $$Excess=\left\{ \begin{array}{ll} Excess-N_{m} & h(k)<\beta -N_{m}\\ Excess-\beta +h(k) & \beta -N_{m}<h(k)<\beta \end{array}\right.$$

In this step, excess pixel counts that exceed the clip limit are recursively distributed among pixels with numbers less than or equal to *β*.

*Step 4*: Cyclic allocation of remaining clipped pixels; if *Excess* is still more than zero after *Step 3*, then the resultant clipped pixels that have not been allocated are redistributed again. If *h*(*k*) < *β*, *then*, *h*(*k*) = *h*(*k*) + 1, *Excess* = *Excess* − 1, and the process is repeated until *Excess* is equal to zero. Thus, the procedure of histogram reassignment is completed and a new histogram with limited contrast is generated. For each region, the grayscale mapping function can be calculated by using Equation (3) in Section 2.1 for its modified histogram.

**Figure 3:**
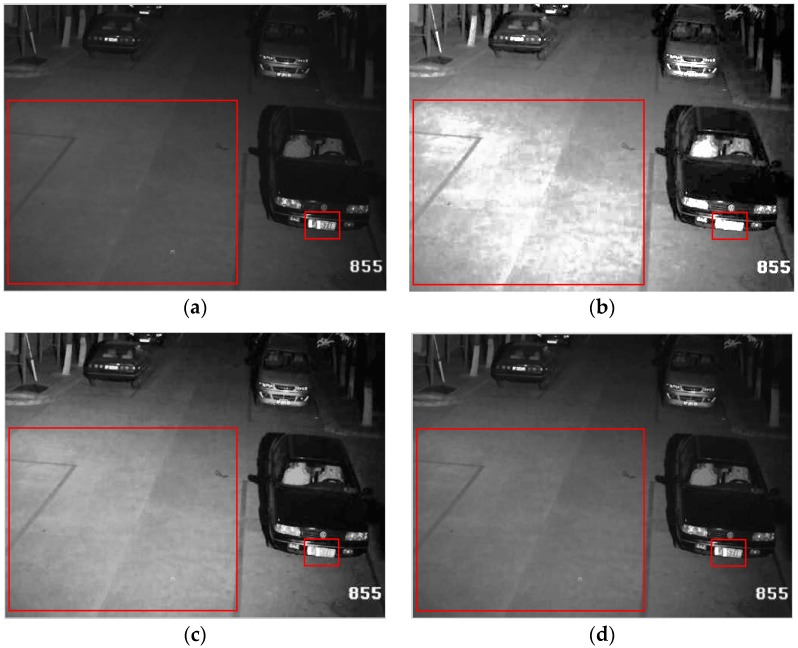
The example of enhanced results by the HE and the clipped HE method. (**a**) Original input image; (**b**) Image enhanced by HE; (**c**) Image enhanced by clipped HE with *β* = 2.5 *N_av_*; (**d**) Image enhanced by clipped HE with *β* = 1.5 *N_av_*.

**Figure 4:**
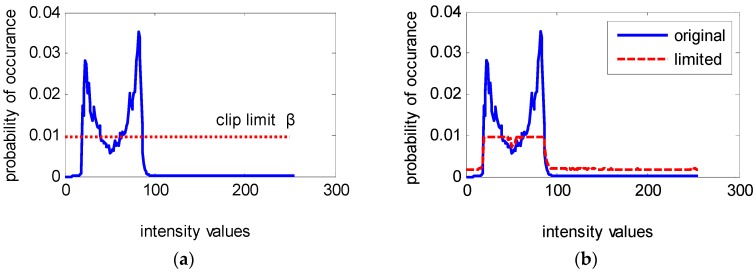

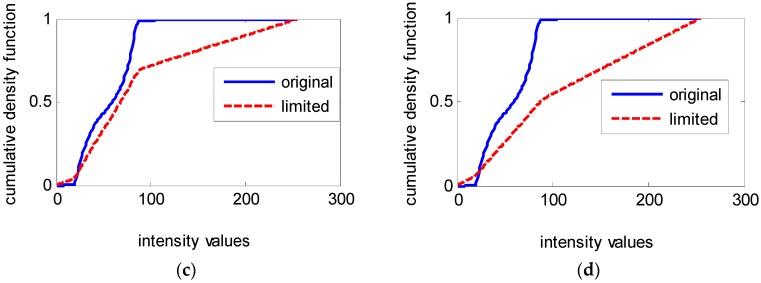
Clipping and redistribution of clipped histogram equalization. (**a**) Histogram of the original input image; (**b**) Histogram of the original input image and the modified histogram after redistribution; (**c**) The cumulative density function of original and modified histograms with *β* = 2.5 *N_av_*; (**d**) The cumulative density function of original and modified histograms with *β* = 1.5 *N_av_*.

Figure 3 shows an example of vehicle surveillance image enhanced results by the HE and the clipped HE method. Figure 3a is the original image with size 500 × 374 pixels, in which most of the pixels are located in the low-level region and it has a dimmed appearance. Figure 3b is the enhanced result by the HE method. To improve overall contrast enhancement of the input image, HE generates a uniform probability distribution of the gray-level by means of expanding the dynamic range of the input image histogram. Unfortunately, over-enhancement occurs in some parts as shown inside the regions of the red rectangle, which result in excessive brightness on the ground and loss of digital information in the license plate in Figure 3b. Figure 3c,d is enhanced images of the clipped HE method with *β* being equal to 2.5 and 1.5 times of the average number of pixels per grayscale *N_av_*, respectively. The over-enhancement phenomenon is avoided to different degrees with different *β* values, as presented in Figure 3c,d. It is clear that the enhanced image quality highly depends on the selection of *β* value, whose value can be chosen empirically. The clipping and redistribution processes are depicted in Figure 4. Figure 4a is the corresponding histogram of Figure 3a, where the horizontal axis and vertical axis represent the intensity values and the probability of occurrence of intensity levels, respectively. Let us assume that the clip limit β be given by the horizontal dotted line. Figure 4b shows the histogram of the original input image in Figure 3a and the modified limited histogram after redistribution according to the clipping and redistribution procedure mentioned above. The comparison curves of the cumulative density function for the original and the modified limited histogram with *β* = 2.5 *N_av_* and *β* = 1.5 *N_av_* are illustrated in Figure 4c,d, respectively. It is obvious that the slope of the clip-limited CDF (dotted line) is less than the original CDF (solid line) in the range of the 20–100 gray-level, in which most pixels for the original image are concentrated. The input gray values in the range of 20–100 are extended to the full range by the original CDF and a narrower range of output values by the clip limited CDF, which is the reason for the excessive enhancement shown in Figure 3b but avoided in Figure 3c,d. Comparing CDF curves in Figure 4c,d, the smaller the clip limit value, the lower the slope of CDF, thus, the higher the ability to restrain the contrast, whereas, higher values of the clip limit result in more contrast enhancement. Figure 4d shows the higher limit ability with a smaller *β*. From the corresponding enhancement image in Figure 3d, it effectively overcomes the over-enhancement problem appeared in Figure 3b, while a very slight over-enhancement phenomenon still occurs with a bigger *β* in Figure 3c. Since this clipped histogram equalization is still a global approach that is useful to enhance the content of the entire input image, it fails to highlight details of the local regions.

### 3.2. Optimum Clip-Limit Strategies

From the discussion in Section 3.1, the major shortcoming of the existing clipped histogram equalization method is that a pre-defined clip limit value is set to limit the input histograms, and in most of the cases the user has to manually select the clip limit to achieve a good enhancement result. This operation makes these methods not appropriate for automatic systems. To enhance the image properly, in this paper, the proposed POSHEOC algorithm employs two measures to find the optimal clip-limit. It selects the optimal clip-limit automatically according to different image data. They are the mean gradient (MG) [45] and mean structural similarity index (MSSIM) [76]. MG is considered as one of the most robust and functionally accurate image quality measures [45,71,72] and is defined by

(14) $$MG=\frac{1}{M\times N}\sum_{i}\sum_{j}\nabla x_{i,j}$$

where $\nabla x_{i,j}$ denotes the image gradient magnitude at pixel (*i*, *j*), which is calculated within a local 3 × 3 square window using the Sobel operators, and *M* × *N* is the image size. In general, MG rises when both the quantity and intensity of gradients of an image increase; a large MG indicates the image has strong local contrast or texture variation, However, MG that is too high is often accompanied by an unnatural look because of over-enhancement and the quality of the image is decreased. Although the mentioned measure can provide a rough estimation of the image contrast, it fails to evaluate the quality under the condition of over-enhanced images. Since in this case, the structure of the original image cannot be preserved, employing some other quantities index that can evaluate the structural similarity between the original and the enhanced images becomes necessary. Hence, MSSIM is employed. It is a quality assessment for measuring the similarity between two images [73,74,76]. Suppose *x* and *y* are two nonnegative image signals for calculating the MSSIM, first we need to calculate SSIM (structural similarity index) [76] using

(15) $$SSIM(x,y)=\frac{\left(2\mu_{x}\mu_{y}+c_{1})(2\sigma_{xy}+c_{2}\right)}{\left(\mu_{x}^{2}+\mu_{y}^{2}+c_{1})(\sigma_{x}^{2}+\sigma_{y}^{2}+c_{2}\right)}$$

where the terms *μ_x_* and *μ*_y_, are the mean intensity, *σ_x_* and *σ_y_* are the standard deviation and *σ_xy_* is the covariance of images *x* and *y*, respectively, and *c*_1_, *c*_2_ are the constant values. The local parameters *μ_x_*, *μ_y_*, *σ_x_*, *σ_y_* and *σ_xy_* are calculated within a local 8 × 8 square window, and the square window slides from pixel to pixel over the whole image. At each step, the SSIM index together with the local parameters are computed within the local window. Based on the value of SSIM, MSSIM can be calculated by

(16) $$MSSIM(X,Y)=\frac{1}{N}\sum_{i=1}^{N}SSIM(x_{i},y_{i})$$

where *X* and *Y* are the input and enhanced image, respectively, *x_i_*, *y_i_* are used to describe the image contents for the *i*th local window; and *N* represents the total number of local windows of the image. In general, a high MSSIM value for an enhanced image represents a good similarity index.

**Figure 5:**
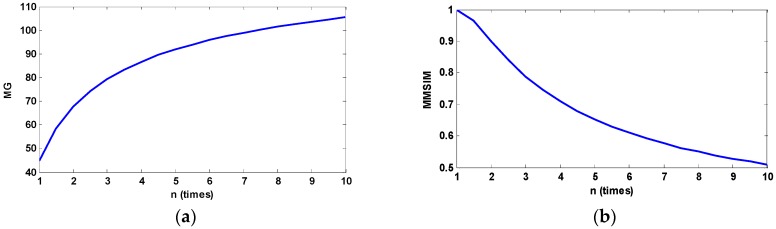
The relationships between the two measures and *n*. (**a**) MG of the enhanced image with different *n* values; (**b**) MMSIM of the enhanced image with different *n* values.

We assume clip limit value *β* = *nN_av_* before performing sub-block equalization in the proposed POSHEOC algorithm according to the analysis in Section 3.1 and the minimum value of *n* is one. It is obvious that the POSHEOC algorithm cannot enhance the image sufficiently if *n* is set to a small value whereas it may result in over-enhancement when *n* is too big; therefore, *n* is set to be in the range of [1, 10]. Figure 5 shows an example of the relationships between the two mentioned measures and *n*. Figure 5a,b plots the metrics MG and MMSIM of enhanced image with different *n*, respectively. Opposite varying tendencies of the two measures with the clip-limit value are observed. Considering the effects of the two factors, finally, we combine the two metrics, and a new index can be built by means of the product of the mean gradient and mean structural similarity (PMGSIM), which is defined by the following Equation (17). It is used to calculate the optimal *n* value. The optimal value of *n* in the range of [1, 10] can be obtained by Equation (18).

(17) PMGSIM(n)=MG(n)×MSSIM(n)

(18) $$n=\underset{n\in [1,10]}{\mathrm{MAX}}\left\{\mathrm{PMGSIM}(n)\right\}$$

**Figure 6:**
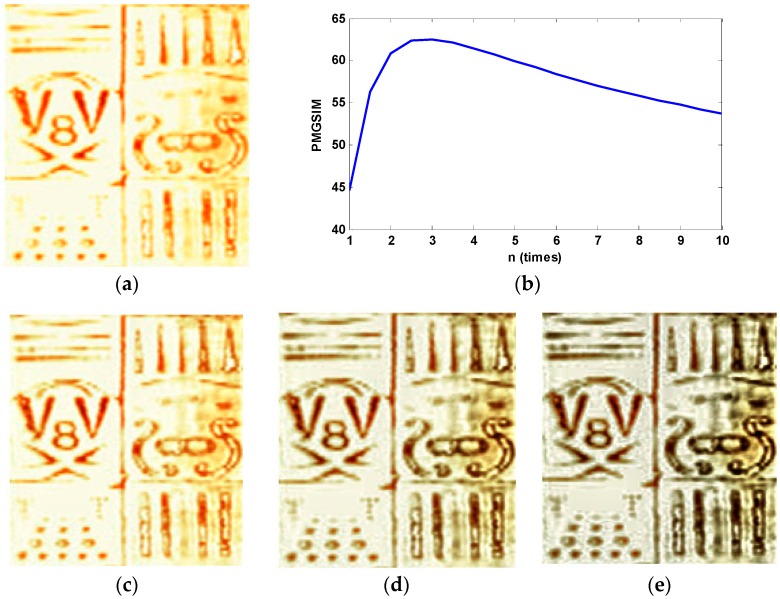
Examples of the proposed POSHEOC with different *n* values. (**a**) The original ultrasonic logging image; (**b**) PMGSIM of the enhanced image with different *n* values; (**c**) The enhanced result of the proposed POSHEOC with *n* = 1.5; (**d**) The enhanced result of the proposed POSHEOC with *n* = 3; (**e**) The enhanced result of the proposed POSHEOC with *n* = 6.

Figure 6 shows an illustration of the selection of the optimal parameter *n*. Figure 6a is the original ultrasonic logging image with size 174 × 187 pixels for the model well, Figure 6b plots the PMGSIM of the enhanced image with different *n* values in terms of Equation (17), and Figure 6c–e shows the enhanced images of POSHEOC with *n* equal to 1.5, 3 and 6, respectively. As can be noticed from Figure 6b, PMGSIM depicts the obvious maximum value with *n* being equal to 3. The corresponding enhanced image yields the highest subjective quality as shown in Figure 6c. It improves the contrast, and reveals the hidden details; e.g., the small hole at the lower left of the image. Figure 6b is the POSHEOC result with *n* = 1.5. It enhances the contrast of original image insufficiently, unable to explore more textural details. In Figure 6c, the POSHEOC result with α = 6 shows a higher contrast but it has a noise addition problem in the homogeneous regions because of over-enhancement. In most cases, the optimal parameter *n* gained using Equation (18) generates good visual quality in extensive experiments.

## 4. Experimental Results and Discussion

In this section, ultrasonic logging images of both the model well and open borehole are utilized to test the performance of the proposed OC POSHE algorithm. The experimental results produced by seven HE and HE-based methods, including HE [62], BBHE [50], RMSHE [51], CLAHE-PL [41], POSHE [63], BOHE [61], MLBOHE [60], are compared with our proposed POSHEOC method.

### 4.1. Subjective Evaluation

**Figure 7:**
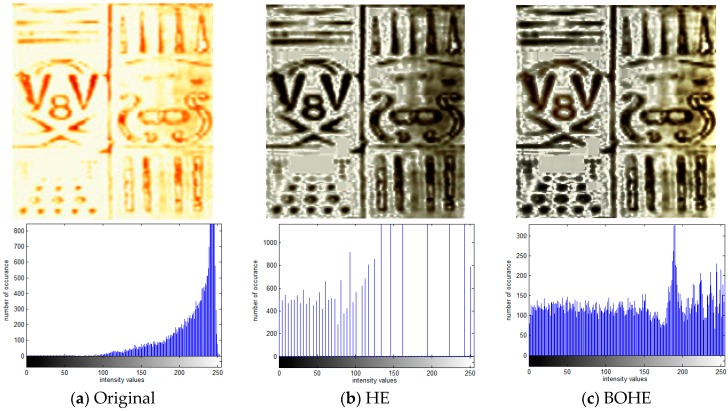

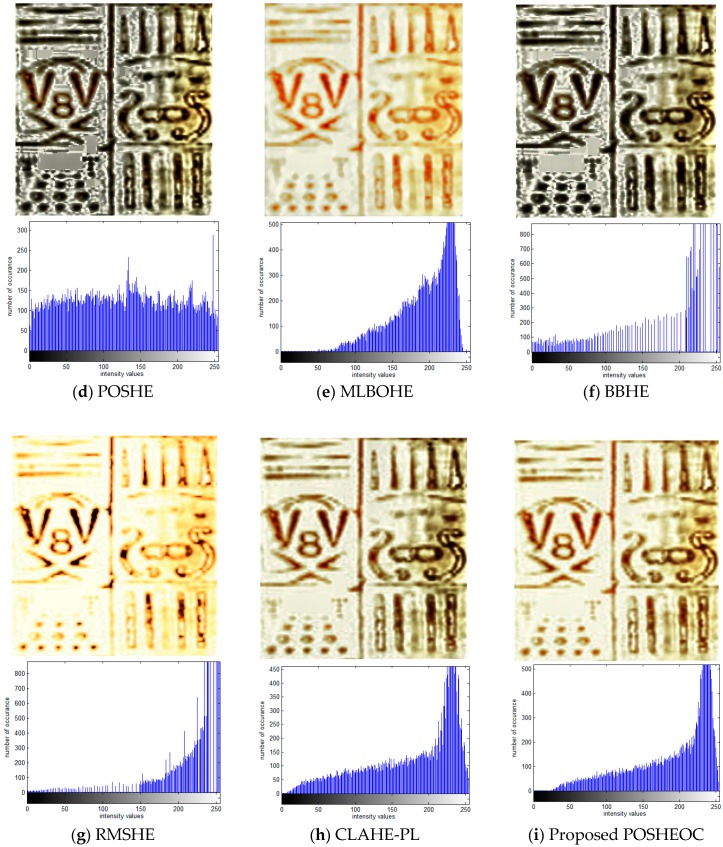
Comparison of enhancement results with corresponding statistical histogram using various techniques for ultrasonic logging image of the model well. (**a**) Original image; (**b**) HE; (**c**) BOHE; (**d**) POSHE; (**e**) MLBOHE; (**f**) BBHE; (**g**) RMSHE; (**h**) CLAHE-PL; (**i**) Proposed POSHEOC.

To demonstrate the performance of the proposed method, we first take the processing results of the model well as an example. Figure 7 shows the processed results of ultrasonic logging image for the model well, which was captured by the ultrasonic image-logging instrument CBIL that uses piezoceramic transducers. The original image and the enhanced images using different enhancement methods along with their corresponding histograms are illustrated in Figure 7a–i. For each histogram, the horizontal axis represents intensity values and the vertical axis corresponds to the number of occurrence of intensity levels. The original image contains the number 8, V shape, arc shape, several circular holes, vertical strips, and some other details. It can be observed from the histogram distribution in Figure 7a that most of the pixels of the original image are concentrated in the high level of intensity. In this case, the image with narrow dynamic range of gray values exhibits low contrast. The HE method directly stretches the histogram of the original image, although the contrast of overall image was enhanced, various undesirable artifacts were produced in the homogeneous background regions due to over enhancement, as indicated in Figure 7b. The over-enhancement phenomenon cannot be avoided in BOHE and POSHE, which also result in unnatural look, as shown in Figure 7c,d. The mentioned three methods increased the contrast and revealed some hidden details. However, the size of some detailed information is amplified along with background noise (see the small holes at the lower left of the image). We notice that the shape of the histograms generated from HE, BOHE, and POSHE are greatly different from their corresponding input histograms. It means that the original brightness is distorted, as can be seen in Figure 7b–d. Figure 7e shows the enhancement results produced by the MLBOHE. This algorithm is actually an advanced version of BOHE, which introduced multiple layers of the block overlapped histogram equalization to successfully overcome noise amplification and intensity distortion problems. Unfortunately, the enhanced image was blurred to some extent because the median filters are adopted to reduce the noise. BBHE slightly preserved the shape of the input histogram, but the middle part is relatively sparse, thus losing some detailed information of intensity. The small holes at the lower left and the arc-shaped at the upper left of the image cannot be displayed completely, as illustrated in Figure 7f. RMSHE further preserved the shape of the input histogram on the basis of BBHE in order to maintain the mean intensity of the original image, yet the contrast was insufficient, and some details hidden in the image are still not brought out, as shown in Figure 7g. Figure 7h is the enhancement result using CLAHE-PL. This technique produces the enhanced image with a good contrast, and the small hole and the arc-shaped located on the above of “digit 8” appeared except that the right half of the image is a slightly dark. Nevertheless, the clip-limit value and power-law transformation parameters in the CLAHE-PL method need to be adjusted to the most appropriate value manually. Figure 7i displays the enhanced image produced by the proposed POSHEOC method. In all experiments, for the input image of size *M* × *N*, the sub-block is set to *M*/8 × *N*/8, the horizontal step size and the vertical step size are both set to *M*/64, by inspection; it produces better visual quality when compared with the previous approaches. The over-enhancement phenomenon is avoided and the contrast is enhanced properly. The small and local details hidden in the image are revealed while the shape of the input histogram is preserved.

**Figure 8:**
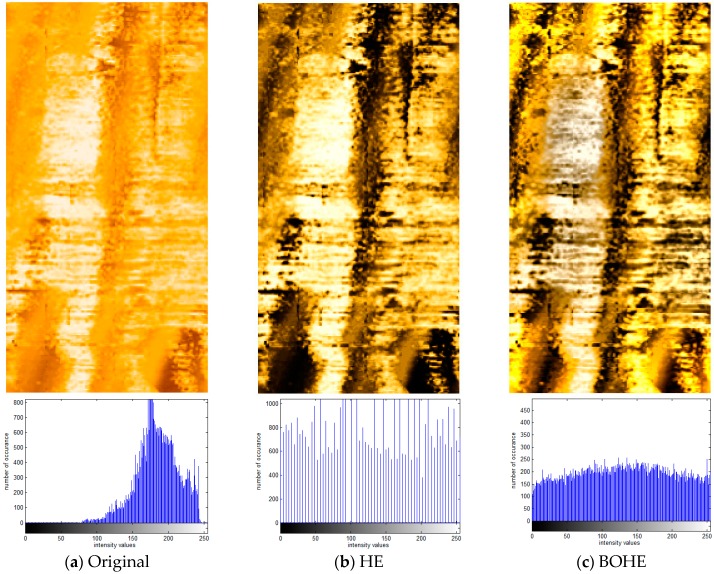

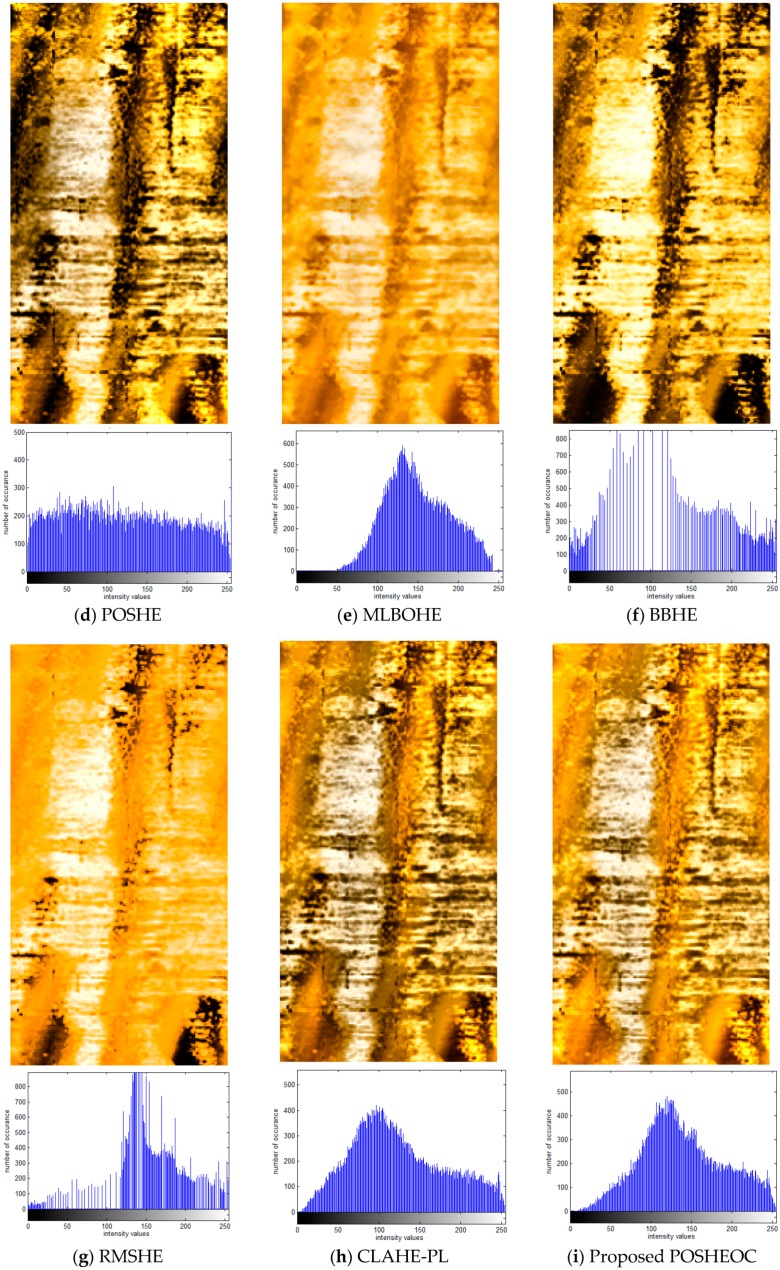
Comparison of enhancement results with corresponding statistical histogram using various techniques for ultrasonic logging image of Changqingyi well. (**a**) Original image; (**b**) HE; (**c**) BOHE; (**d**) POSHE; (**e**) MLBOHE; (**f**) BBHE; (**g**) RMSHE; (**h**) CLAHE-PL; (**i**) Proposed POSHEOC.

Figure 8 shows an example of the processed results of the ultrasonic logging image for Changqingyi well, which was captured by the piezoceramic ultrasonic imaging logging instrument BHTV. Figure 8a illustrates the original amplitude image with size 161 × 312 pixels for the Changqingyi well and its corresponding histogram, and, as can be seen, it exhibits low contrast which is not conducive to the analysis and recognition of geological features. In Figure 8b, HE is used directly to equalize the input histogram for generating an enhanced image with high contrast; the histogram seems to be relatively sparse. This is due to the fact that some pixels with low probability of occurrence are merged, which leads to the fact that bright regions of the middle part in the image are submerged. It also makes the regions with low intensity even darker and excessively bright in high gray level regions because of excessive contrast enhancement. Although the enhancement results of both the BOHE and POSHE methods highlight the local detailed information, the regions with over-enhancement still appear. In particular, the regions with relatively low intensity are changed into large areas of dark regions and the mean brightness is heavily shifted, leading to an unnatural look. Similar to the histogram distribution in Figure 8c,d, they are very different from the corresponding input histogram. MLBOHE significantly preserves the shape of the input histogram and removes the background noise. However, it produces an obscure enhanced image, as indicated in Figure 8e. The result of the BBHE is similar to that of the HE. It enhances overall contrast while missing local details, as shown in Figure 8f. RMSHE preserves the mean brightness of the original input image to some extent by iteratively applying BBHE to the sub-histograms. The local details are still not clear, as displayed in Figure 8g. In Figure 8h, CLAHE obtains a good enhancement without over-enhancement, and local details hidden in the bright area appear. Figure 8i shows the enhanced image using the proposed POSHEOC method. It generates a good visual quality without unnatural artifacts, and the contrast is enhanced properly. Local details hidden in the bright regions are revealed and the horizontal textures located on the lower half part of the image are more distinguishable. Although the enhanced results between the CLAHE-PL and POSHEOC approaches are competitive, it can be observed that the latter has better ability to preserve the shape of the input histogram. Therefore, the proposed POSHEOC method is more effective than all the previous techniques. Advantages in objective metrics will be provided in the next subsection.

**Figure 9:**
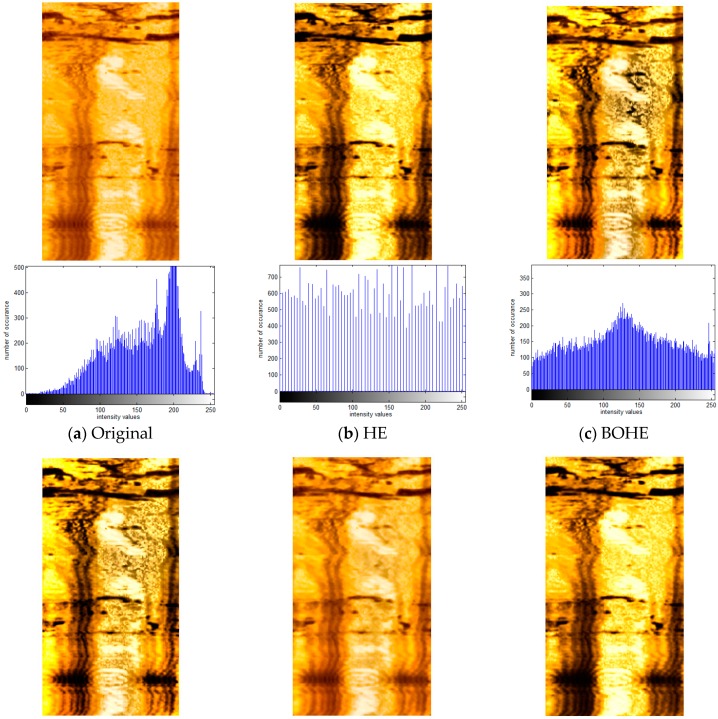

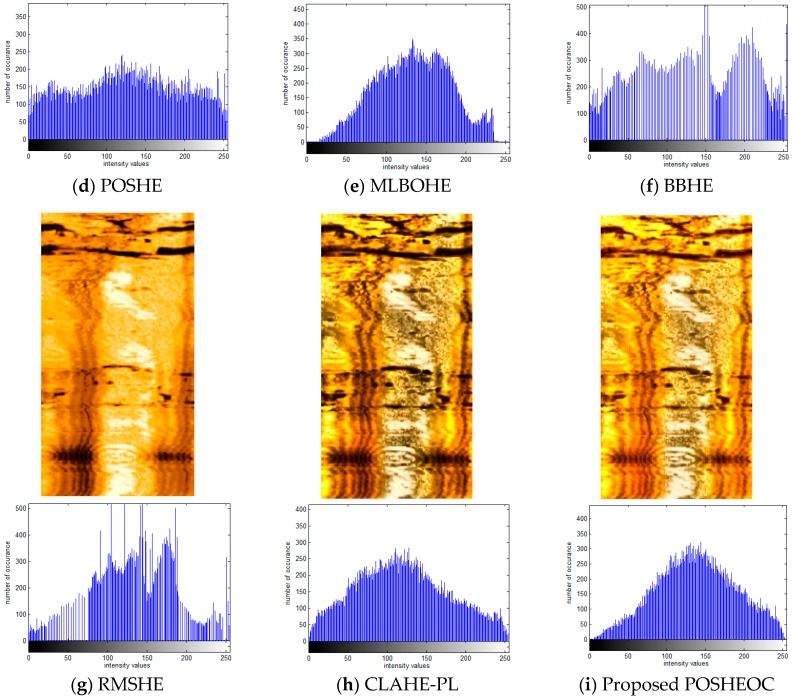
Comparison of enhancement results with corresponding statistical histogram using various techniques for ultrasonic logging image of Changqingli well. (**a**) Original image; (**b**) HE; (**c**) BOHE; (**d**) POSHE; (**e**) MLBOHE; (**f**) BBHE; (**g**) RMSHE; (**h**) CLAHE-PL; (**i**) Proposed POSHEOC.

Figure 9 shows another example of the processed results of the ultrasonic logging image for Changqingli well and it was captured by the ultrasonic imaging logging instrument CBIL. Figure 9a is the original amplitude image with fracture information and its corresponding histogram, while Figure 9b–i demonstrate enhanced images with different methods, and the size of the test image is 144 × 270. It is observed from the histogram distribution of Figure 9a that the contrast of the whole input image is better than that of the previous two examples; however, the fractured belts in the middle part of the image are still in low contrast. Although the contrast of the whole image is significantly increased by the HE, BOHE, POSHE and BBHE methods and the image of the fractured belts is clearer than the original one, some adverse effects were generated. For example, the left and right sides of the lower half part in the image become too dark, as shown in Figure 9b–d,f. Similar to the previous examples in Figure 7 and Figure 8, MLBOHE eliminated the background noise but blurred the image, as displayed in Figure 9e. Obviously, the contrast of the whole image along with fractured belts is not well enhanced by the RMSHE, as shown in Figure 9g. CLAHE-PL and the proposed POSHEOC obtained a good enhancement results without over-enhancement, but the two methods have better ability of brightness preservation and produce fewer artifacts as demonstrated by Figure 9h,i.

### 4.2. Objective Evaluation

In addition to the subjective evaluation, quantitative measures are also crucial in comparing the performance of different image enhancement approaches. In this paper, the product of mean gradient and mean structural similarity (PMGSIM) given by Equation (17), the peak signal to noise ratio (PSNR) [59,60,74], the information entropy (IE) [53,68], the absolute mean brightness error (AMBE) [59,60,74], and the local contrast (LC) [75] values are employed here for the objective evaluation. Assuming that the size of the original image *X* is *M* × *N*, and *Y* is the enhanced image. PSNR is then defined as,

(19) $$PSNR=10\log\left[\frac{N\times Mf_{\max}^{2}}{\sum_{i=1}^{N}\sum_{j=1}^{M}{\left[X(i,j)-Y(i,j)\right]}^{2}}\right]$$

where *f_max_* is the maximum intensity of the input image, for the common 8-bit gray-level image with 256 possible gray level values, as it is known that *f_max_* = 255. Generally, PSNR is used to estimate the artifacts or noise produced in the process of contrast enhancement. It is expected that a good enhancement method will generate a high PSNR value. AMBE is the absolute mean brightness error between the input and output image, which is described as:

(20) $$\mathrm{AMBE}=\left|\mu_{X}-\mu_{Y}\right|$$

where, *μ_X_* and *μ_Y_* are the mean intensity values of *X* and *Y*, respectively. The lower the value of AMBE, the better is the brightness preservation and vice versa. IE is an effective way to evaluate the amount of information content within an image; for the image with the gray level in the range [0, *L* − 1], the entropy of the image can be expressed as

(21) $$\mathrm{IE}=-\sum_{i=0}^{L-1}p(s_{i}^{})\log_{2}^{}p(s_{i})$$

where *p*(*s_i_*) represents probability density function for a given image at gray level *s_i_.* In general, a larger value of the entropy indicates more richness of details is available in the image. The local contrast criterion is defined in Equation (22)

(22) $$\mathrm{LC}=\frac{1}{MN}\sum_{i=1}^{M}\sum_{j=1}^{N}\frac{I_{i,j}^{max}-I_{i,j}^{min}}{I_{i,j}^{max}+I_{i,j}^{min}+c}$$

where $I_{i,j}^{\max}$ and $I_{i,j}^{\min}$ are the maximum and minimum gray values of a specific block (5 × 5 in this work) center on pixel (*i*, *j*), and *c* is a small constant with the value equals to 0.0001 to avoid dividing by 0. A large local contrast value represents a strong contrast.

Table 1 lists the corresponding quantitative measures PMGSIM, PSNR, IE, AMBE and LC results of different enhancement algorithms for ultrasonic logging images of the model well in Figure 7. The proposed POSHEOC produces the highest PMGSIM values when compared to all the other techniques, since the proposed POSHEOC enhances the contrast of image effectively while taking into account of the negative impacts of over-enhancement. Comparison with PSNR, AMBE shows that the performance of the proposed POSHEOC method on the image is better than the other six methods except for RMSHE. In the process of RMSHE, the output mean is closer to the input mean as the recursion level becomes larger, and the original mean brightness can be preserved to reduce artifacts at a greater degree through increasing the level of histogram division. Therefore, it obtains the highest PSNR and lowest AMBE values; however, its contrast enhancement capability was not notable, as can be noticed from the LC value in Table 1, resulting in poor quality. By inspection, the IE and LC values of BOHE, POSHE are relatively higher than the other methods. Since an image with uniform distribution histogram has the maximum information entropy, a high value of IE owing to the gray level is evenly distributed in POSHE and BOHE, as can be seen from histogram distribution in Figure 7c–d. However, the values of PMGSIM, PSNR, AMBE for BOHE, POSHE show the poorer performance among the tested methods. This is due to more emphasis on local details which also results in a high LC value; those coarse details are the results from background noise amplification. Therefore, the higher IE and LC values of BOHE, POSHE do not represent better quality; we can observe enhanced results accompanied by undesired artifacts in Figure 7c–d. A median value of IE and LC for the proposed POSHEOC obtains better quality, since the very high and very low value of the two aforementioned indexes represents excessive enhancement and insufficient enhancement, respectively. Further, when visual quality between the CLAHE-PL and the proposed POSHEOC are almost the same, all the objective metrics of the proposed POSHEOC are better than those of CLAHE-PL except for the slightly higher IE value of CLAHE-PL. Table 2 and Table 3 summarize corresponding performance measure values of PMGSIM, PSNR, IE, AMBE and LC for ultrasonic logging image of Changqingli well in Figure 8 and Figure 9 using eight different methods. The variation tendency of simulation data is almost in accordance with that of data in Table 1. The proposed algorithm is demonstrated to have the best enhancement effect when compared with other existing approaches from Table 2 and Table 3.

## 5. Conclusions

In this work, a novel POSHE-based optimum clip-limit contrast enhancement method to improve the ultrasonic well logging images (POSHEOC) is proposed. The algorithm introduces the idea of contrast-limitation to modify the cumulative distribution functions of the partially overlapped sub-block histogram equalization and obtains optimal clip-limit by considering the effects of the mean gradient and mean structural similarity. The proposed algorithm makes the choice of optimal clip-limit automatically according to the different input image. The proposed POSHEOC shows better performance in enhancing the contrast, emphasizing the local details while preserving the brightness and restricting the excessive enhancement compared with several other histogram equalization-based techniques from the literature. This is demonstrated in experimental results based on visual perceptual evaluation and quantitative measures. We anticipate that this study also helps to provide a feasible and efficient approach to improve well logging images and is significant for the interpretation of actual ultrasonic logging data. In addition, future research will involve the development of simpler methods to obtain the optimal clip-limit value.

## Figures and Tables

**Table 1:** Quantitative results for ultrasonic logging image of model well with various methods.

Methods	Objective Indexes				
	PMGSIM	PSNR	IE	AMBE	LC
HE	41.1477	7.8129	5.2915	91.3723	0.3517
BOHE	32.7773	7.1055	7.9546	97.4517	0.5681
POSHE	41.5468	7.7988	7.9259	91.5251	0.6086
MLBOHE	45.2901	15.7386	6.9873	38.0596	0.1769
BBHE	55.4418	14.5983	5.9073	25.1141	0.3563
RMSHE	52.2859	25.1331	5.9667	1.7287	0.1913
CLAHE-PL	60.1273	13.6930	7.4505	41.4485	0.2067
POSHEOC	62.5286	16.6169	7.2052	27.7857	0.2773

**Table 2:** Quantitative results for ultrasonic logging image of Changqingyi well with various methods.

Methods	Objective Indexes				
	PMGSIM	PSNR	IE	AMBE	LC
HE	52.4388	13.7409	5.8441	31.0693	0.2991
BOHE	48.6238	10.8948	7.9774	54.0931	0.5068
POSHE	54.3196	12.7994	7.9665	38.2192	0.5249
MLBOHE	40.7913	16.7165	7.2906	34.4128	0.1624
BBHE	52.9611	14.4309	6.8055	24.2409	0.4237
RMSHE	47.6993	24.7405	6.7605	2.7983	0.2098
CLAHE-PL	58.4960	14.5198	7.7645	35.4124	0.2783
POSHEOC	61.1664	17.2683	7.6481	19.6878	0.3606

**Table 3:** Quantitative results for ultrasonic logging image of Changqingli well with various methods.

Methods	Objective Indexes				
	PMGSIM	PSNR	IE	AMBE	LC
HE	49.2776	17.8113	5.9706	8.2204	0.2409
BOHE	53.4988	14.5454	7.9473	29.0144	0.4281
POSHE	54.6502	16.6845	7.9561	8.4551	0.2990
MLBOHE	40.1781	19.3844	7.5268	24.8180	0.1898
BBHE	49.7495	18.2670	7.2280	4.1289	0.3378
RMSHE	44.0206	29.3174	7.2733	0.7827	0.2234
CLAHE-PL	56.4173	16.8092	7.8462	16.0812	0.2857
POSHEOC	58.2492	18.5768	7.6870	2.3548	0.3450

## References

[1] Lin B., Giurgiutiu V. (2006). Modeling and testing of PZT and PVDF piezoelectric wafer active sensors. Smart Mater. Struct..

[2] Lu G., Feng Q., Li Y., Wang H., Song G. (2017). Characterization of ultrasound energy diffusion due to small-size damage on an aluminum plate using piezoceramic transducers. Sensors.

[3] Yin H., Wang T., Yang D., Liu S., Shao J., Li Y. (2016). A smart washer for bolt looseness monitoring based on piezoelectric active sensing method. Appl. Sci..

[4] Xu K., Kong Q., Chen S., Song G. (2017). Early Determination of the Presence of Low Strength Concrete in Reinforced Concrete Beam-Column Joints Using Piezoceramic-Based Transducers. IEEE Sens. J..

[5] Zhang J., Li Y., Du G., Song G. (2018). Damage detection of l-shaped concrete filled steel tube (L-CFST) columns under cyclic loading using embedded piezoceramic transducers. Sensors.

[6] Lynch J.P., Loh K.J. (2006). A summary review of wireless sensors and sensor networks for structural health monitoring. Shock Vib. Dig..

[7] Yang Y., Divsholi B.S., Soh C.K. (2010). A reusable PZT transducer for monitoring initial hydration and structural health of concrete. Sensors.

[8] Song G., Wang C., Wang B. (2017). Structural Health Monitoring (SHM) of Civil Structures. Appl. Sci..

[9] Yang Y., Annamdas V.G.M., Wang C., Zhou Y. (2008). Application of multiplexed FBG and PZT impedance sensors for health monitoring of rocks. Sensors.

[10] Kim S.D., In C.W., Cronin K.E., Sohn H., Harries K. (2007). Reference-free NDT technique for debonding detection in CFRP-strengthened RC structures. J. Struct. Div..

[11] Mańka M., Rosiek M., Martowicz A., Stepinski T., Uhl T. (2016). PZT based tunable Interdigital Transducer for Lamb waves based NDT and SHM. Mech. Syst. Signal. Process..

[12] Gao W., Zhang G., Li H., Huo L., Song G. (2018). A novel time reversal sub-group imaging method with noise suppression for damage detection of plate-like structures. Struct. Control Health Monit..

[13] Luo M., Li W., Hei C., Song G. (2016). Concrete infill monitoring in concrete-filled FRP tubes using a PZT-based ultrasonic time-of-flight method. Sensors.

[14] Song G., Gu H., Mo Y.L., Hsu T.T.C., Dhonde H. (2007). Concrete structural health monitoring using embedded piezoceramic transducers. Smart Mater. Struct..

[15] Kong Q., Robert R.H., Silva P., Mo Y.L. (2016). Cyclic crack monitoring of a reinforced concrete column under simulated pseudo-dynamic loading using piezoceramic-based smart aggregates. Appl. Sci..

[16] Xu J., Hao J., Li H., Luo M., Guo W., Li W. (2017). Experimental Damage Identification of a Model Reticulated Shell. Appl. Sci..

[17] Sun F.P., Chaudhry Z., Liang C., Rogers C.A. (1997). Truss structure integrity identification using PZT sensor-actuator. NDT E Int..

[18] Du G., Kong Q., Zhou H., Gu H. (2017). Multiple cracks detection in pipeline using damage index matrix based on piezoceramic transducer-enabled stress wave propagation. Sensors.

[19] Zhu J., Ren L., Ho S.C., Jia Z., Song G. (2017). Gas pipeline leakage detection based on PZT sensors. Smart Mater. Struct..

[20] Du G., Kong Q., Wu F., Ruan J., Song G. (2016). An experimental feasibility study of pipeline corrosion pit detection using a piezoceramic time reversal mirror. Smart Mater. Struct..

[21] Venugopal V.P., Wang G. (2015). Modeling and analysis of Lamb wave propagation in a beam under lead zirconate titanate actuation and sensing. J. Intell. Mater. Syst. Struct..

[22] Kong Q., Hou S., Ji Q., Mo Y.L., Song G. (2013). Very early age concrete hydration characterization monitoring using piezoceramic based smart aggregates. Smart Mater. Struct..

[23] Tian Z., Huo L., Gao W., Li H., Song G. (2017). Modeling of the attenuation of stress waves in concrete based on the Rayleigh damping model using time-reversal and PZT transducers. Smart Mater. Struct..

[24] Gao W., Huo L., Li H., Song G. (2018). Smart concrete slabs with embedded tubular PZT transducers for damage detection. Smart Mater. Struct..

[25] Lu G., Li Y., Wang T., Xiao H., Song G. (2017). A multi-delay-and-sum imaging algorithm for damage detection using piezoceramic transducers. J. Intell. Mater. Syst. Struct..

[26] Lu G., Li Y., Zhou M., Feng Q., Song G. (2018). Detecting Damage Size and Shape in a Plate Structure Using PZT Transducer Array. J. Aerosp. Eng..

[27] Zhang G., Gao W., Song G., Song Y. (2016). An imaging algorithm for damage detection with dispersion compensation using piezoceramic induced lamb waves. Smart Mater. Struct..

[28] Gao W., Huo L., Li H., Song G. (2018). An Embedded Tubular PZT Transducer Based Damage Imaging Method for Two-Dimensional Concrete Structures. IEEE Access.

[29] Valente S.A., Zibetti M.V.W., Pipa D.R., Maia J.M., Schneider F.K. (2017). An Assessment of Iterative Reconstruction Methods for Sparse Ultrasound Imaging. Sensors.

[30] Gang T., Hu M., Bai X., Rong Q. (2018). Sensitivity-Improved Ultrasonic Sensor for 3D Imaging of Seismic Physical Model Using a Compact Microcavity. Sensors.

[31] Birchak J.R., Linyaev E., Robbins C.A., Roessler D.E., Halliburton Co. (1997). Acoustic Transducer for LWD Tool. U.S. Patent.

[32] Aron J.B., Chang S.K., Klasel D.A., Lau T.M., Schlumberger Technology Corp (1998). Transducer for Sonic Logging-While-Drilling. U.S. Patent.

[33] Yogeswaren E., PathFinder Energy Services Inc. (2006). Acoustic Sensor for Downhole Measurement Tool. U.S. Patent.

[34] Massiot C., Mcnamara D.D., Lewis B. (2015). Processing and analysis of high temperature geothermal acoustic borehole image logs in the Taupo Volcanic zone, New Zealand. Geothermics.

[35] Lai J., Wang G., Wang S., Cao J., Li M., Pang X. (2018). A review on the applications of image logs in structural analysis and sedimentary characterization. Mar. Pet. Geol..

[36] Zohreh M., Junin R., Jeffreys P. (2014). Evaluate the borehole condition to reduce drilling risk and avoid potential well bore damages by using image logs. J. Pet. Sci. Eng..

[37] Sun Z., Chen H., Liu X. (2013). Case studies of casing inspection with multi-functional ultrasonic imaging logging tool. J. Acoust. Soc. Am..

[38] Liang M., Peng S., Du W., Lu Y. (2018). Tectonic stress estimation from ultrasonic borehole image logs in a coal bed methane well, northeastern Qinshui Basin, China. J. Nat. Gas Sci. Eng..

[39] Xiao K., Zou C., Xiang B., Yue X., Zhou X., Li J., Zhao B. (2014). Log response of ultrasonic imaging and its significance for deep mineral prospecting of scientific drilling borehole-2 in Nanling district, China. J. Geophys. Eng..

[40] Zhang J., Nie X., Xiao S., Zhang C., Zhang C., Zhang Z. (2018). Generating porosity spectrum of carbonate reservoirs using ultrasonic imaging log. Acta Geophys..

[41] Fu Q., Celenk M., Wu A. (2018). An improved algorithm based on CLAHE for ultrasonic well logging image enhancement. Clust. Comput..

[42] Tu J., Yu H., Li C., Zou W. (2011). Study of Histogram Equalization for Ultrasonic Logging Well Image. Video Eng..

[43] Jeong C.K., Baek C., Kingon A.I., Park K.I., Kim S.H. (2018). Lead-free perovskite nanowire-employed piezopolymer for highly efficient flexible nanocomposite energy harvester. Small.

[44] Che X.-H., Qiao W.-X., Ju X.-D., Wang R.-J. (2016). Azimuthal cement evaluation with an acoustic phased-arc array transmitter: Numerical simulations and field tests. Appl. Geophys..

[45] Liu J., Zhou C., Chen P., Kang C. (2017). An Efficient Contrast Enhancement Method for Remote Sensing Images. IEEE Geosci. Remote Sens. Lett..

[46] Lai Y.R., Tsai P.C., Yao C.Y., Ruan S.J. (2017). Improved local histogram equalization with gradient-based weighting process for edge preservation. Multimed. Tools Appl..

[47] Ibrahim H., Kong N.S.P. (2007). Brightness Preserving Dynamic Histogram Equalization for Image Contrast Enhancement. IEEE Trans. Consum. Electron..

[48] Huang L., Zhao W., Wang J., Sun Z. (2015). Combination of contrast limited adaptive histogram equalisation and discrete wavelet transform for image enhancement. IET Image Process..

[49] Chiu C.C., Ting C.C. (2016). Contrast Enhancement Algorithm Based on Gap Adjustment for Histogram Equalization. Sensors.

[50] Kim Y.T. (1997). Contrast enhancement using brightness preserving bi-histogram equalization. IEEE Trans. Consum. Electron..

[51] Chen S.D., Ramli A.R. (2003). Contrast enhancement using recursive mean-separate histogram equalization for scalable brightness preservation. IEEE Trans. Consum. Electron..

[52] Chen S.D., Ramli A.R. (2004). Minimum mean brightness error bi-histogram equalization in contrast enhancement. IEEE Trans. Consum. Electron..

[53] Singh K., Kapoor R. (2014). Image enhancement using exposure based sub image histogram equalization. Pattern Recognit. Lett..

[54] Singh K., Kapoor R., Sinha S.K. (2015). Enhancement of low exposure images via recursive histogram equalization algorithms. Optik.

[55] Parihar A.S., Verma O.P. (2017). Contrast enhancement using entropy-based dynamic sub-histogram equalisation. IET Image Process..

[56] Abdullah-Al-Wadud M., Kabir M.H., Dewan M.A.A., Chae O. (2007). A Dynamic Histogram Equalization for Image Contrast Enhancement. IEEE Trans. Consum. Electron..

[57] Huang S.C., Cheng F.C., Chiu Y.S. (2013). Efficient contrast enhancement using adaptive gamma correction with weighting distribution. IEEE Trans. Image Process..

[58] Arici T., Dikbas S., Altunbasak Y. (2009). A Histogram Modification Framework and Its Application for Image Contrast Enhancement. IEEE Trans. Image Process..

[59] Zhao Q., Huang L. (2015). Brightness preserving image enhancement based on a gradient and intensity histogram. J. Electron. Imaging.

[60] Kong N.S.P., Ibrahim H. (2011). Multiple layers block overlapped histogram equalization for local content emphasis. Comput. Electr. Eng..

[61] Kim T.K., Paik J.K., Kang B.S. (1998). Contrast enhancement system using spatially adaptive histogram equalization with temporal filtering. IEEE Trans. Consum. Electron..

[62] Gonzalez R.C., Woods R.E. (2007). Digital Image Processing.

[63] Kim J.Y., Kim L.S., Hwang S.H. (2001). An advanced contrast enhancement using partially overlapped sub-block histogram equalization. IEEE Trans. Circuits Syst. Video Technol..

[64] Reza A.M. (2004). Realization of the Contrast Limited Adaptive Histogram Equalization (CLAHE) for Real-Time Image Enhancement. J. VLSI Signal Process. Syst. Signal Image Video Technol..

[65] Zuiderveld K. (1994). Contrast Limited Adaptive Histogram Equalization.

[66] Yan J.P., Shou X.Y., Shao Z.P., Yao S.X., Zhao Z.M. (2006). The Method of Image Dynamic Intensify and Morphing in Imaging Log. Well Logging Technol..

[67] Wang X., Peng T., Lei G., Zhang J., Zhao X. (2015). On the Method of XRMI Dynamic Enhancement and Full Borehole Imaging and Its Application. Well Logging Technol..

[68] Jenifer S., Parasuraman S., Kadirvelu A. (2016). Contrast enhancement and brightness preserving of digital mammograms using fuzzy clipped contrast-limited adaptive histogram equalization algorithm. Appl. Soft Comput..

[69] Chen H.O., Kong N.S.P., Ibrahim H. (2010). Bi-histogram equalization with a plateau limit for digital image enhancement. IEEE Trans. Consum. Electron..

[70] Al-Ameen Z., Sulong G., Rehman A., Al-Dhelaan A., Saba T., Al-Rodhaan M. (2015). An innovative technique for contrast enhancement of computed tomography images using normalized gamma-corrected contrast-limited adaptive histogram equalization. Eurasip J. Adv. Signal Process..

[71] Jiao L., Sun Z., Sha A. Improvement of Image Contrast with Local Adaptation. Proceedings of the 2010 Second International Conference on Multimedia and Information Technology.

[72] Liu Y.F., Guo J.M., Lai B.S. (2016). Parametric-Oriented Fitting for Local Contrast Enhancement. Inform. Sci..

[73] Gupta B., Agarwal T.K. (2017). Linearly quantile separated weighted dynamic histogram equalization for contrast enhancement. Comput. Electr. Eng..

[74] Joseph J., Jayaraman S., Periyasamy R., Simi V.R. (2017). An objective method to identify optimum clip-limit and histogram specification of contrast limited adaptive histogram equalization for MR images. Biocybern. Biomed. Eng..

[75] Wang Y., Pan Z. (2017). Image contrast enhancement using adjacent-blocks-based modification for local histogram equalization. Infrared Phys. Technol..

[76] Wang Z., Bovik A.C., Sheikh H.R., Simoncelli E.P. (2004). Image quality assessment: From error visibility to structural similarity. IEEE Trans. Image Process..

